# Identification of Liver Fibrosis-Related MicroRNAs in Human Primary Hepatic Stellate Cells Using High-Throughput Sequencing

**DOI:** 10.3390/genes13122201

**Published:** 2022-11-24

**Authors:** Xu Liu, Heming Ma, Ruihong Wu, Huan Wang, Hongqin Xu, Shuxuan Li, Guangyi Wang, Guoyue Lv, Junqi Niu

**Affiliations:** 1 Department of Hepatology, First Hospital of Jilin University, Changchun 130021, China; 2 Center of Infectious Diseases and Pathogen Biology, First Hospital of Jilin University, Changchun 130021, China; 3 Department of Infectious Diseases, Union Hospital, Tongji Medical College, Huazhong University of Science and Technology, Wuhan 430022, China; 4 Department of Hepatobiliary and Pancreatic Surgery, First Hospital of Jilin University, Changchun 130021, China

**Keywords:** human primary hepatic stellate cell, liver fibrosis, microRNA, next-generation sequencing, target

## Abstract

MicroRNAs (miRNAs) participate in hepatic stellate cell (HSC) activation, which drives liver fibrosis initiation and progression. We aimed to identify novel hepatic fibrosis targets using miRNA sequencing (miRNA-seq) of human primary HSCs. Surgically resected liver tissues were used to extract HSCs. Based on next-generation sequencing, miRNA-seq was performed on four pairs of HSCs before and after in vitro culture. Additionally, we compared our data with open access miRNA-seq data derived from fourteen cirrhotic and nine healthy liver tissues. Selected miRNAs associated with fibrosis were verified by quantitative real-time PCR. Target mRNAs of differentially expressed (DE) miRNAs were predicted to construct co-expression networks. We identified 230 DEmiRNAs (118 upregulated and 112 downregulated) upon HSC activation. Of the 17 miRNAs with the most significant differences in expression, liver disease-related miRNAs included *miR-758-3p*, *miR-493-5p*, *miR-409-3p*, *miR-31-5p*, *miR-1268a*, and *miR-381-3p*, which might play roles in hepatic fibrosis. Moreover, *let-7g-5p*, *miR-107*, *miR-122-5p*, *miR-127-3p*, *miR-139-5p*, *miR-148a-3p*, *miR-194-5p*, *miR-215-5p*, *miR-26a-5p*, *miR-340-5p*, *miR-451a*, and *miR-99a-5p* were common between our data and the publicly available sequencing data. A co-expression network comprising 1891 matched miRNA–mRNA pairs representing 138 DEmiRNAs and 1414 DEmRNAs was constructed. *MiR-1268a* and *miR-665*, possessing the richest target DEmRNAs, may be vital in HSC activation. The targeted genes were involved in collagen metabolism, extracellular matrix structural constituent, cytoskeletal protein binding, and cell adhesion. The miRNAs we identified may provide a basis and reference for the selection of diagnostic and therapeutic targets for hepatic fibrosis.

## 1. Introduction

Hepatic fibrosis is a pathological repair response characterized by diffuse over-deposition and abnormal distribution of the extracellular matrix (ECM) [1,2]. Hepatitis B virus, hepatitis C virus, alcohol, and high-fat diets are the most common etiologies of cirrhosis [3]. Epidemiologically, cirrhosis causes approximately 1.2 million deaths worldwide each year, leading to an increase in healthcare utilization and presenting a major social burden [4]. Hepatic stellate cells (HSCs) constitute the main ECM-producing cell population in the liver [5]. HSC activation, proliferation, and transformation play pivotal roles in the process of hepatic fibrosis. Consequently, elucidation of the molecular mechanisms of HSC activation could be crucial for identifying early diagnostic and therapeutic targets of liver fibrosis.

MicroRNAs (miRNAs) are approximately 22-nucleotide-long single-stranded non-coding RNAs [6], accounting for 1–2% of all mammalian genes. They are expressed in a tissue-specific and time-sequential fashion, and have been implicated in the regulation of several mRNAs through miRNA response elements. A single miRNA can affect tens to hundreds of different transcripts by inducing post-transcriptional effects on mRNA stability and translation [7,8]. More than 60% of coding genes contain miRNA target sequences, and nearly 1/3 of human genes can be regulated by miRNAs [9,10,11]. Dysregulation of endogenous miRNAs may underlie a variety of human diseases, including infections, cancer, and vascular and nervous system diseases [12,13,14,15].

Recent studies have suggested that divergent miRNAs are involved in the development of hepatic fibrosis and activation of HSCs. Numerous miRNAs have been associated with liver fibrogenesis [6,16,17]. For example, the overexpression of the *miR-199* or *miR-200* families was positively correlated with the activation of the transforming growth factor-β (TGF-β)/Smad signaling pathway [18,19]. In contrast, *miR-150* and *miR-194* inhibited the activation of HSCs and production of ECM to a certain extent by inhibiting the expression of C-myb and RAC1 [20]. Additionally, serological concentrations of *miR-122* and *miR-29a* can be used to monitor the progression of liver fibrosis [21]. *MiR-21* promoted the progression of fibrosis in multiple organs [22,23,24]; however, its role in fibrogenesis is debatable. Caviglia et al. [25] suggested that *miR-21* does not influence fibrosis or hepatocellular carcinoma (HCC) formation.

Several studies have analyzed miRNA expression profiles of HSCs using microarray technology [26,27,28], mostly using animal models. However, the influence of species-related differences can be avoided using human-derived primary cells, and high-throughput sequencing has gradually replaced microarrays in recent years [29].

To this end, we attempted to identify the potential genes associated with hepatic fibrosis using microRNA sequencing (miRNA-seq) of human primary HSCs based on next-generation sequencing (NGS) technology, in order to determine novel strategies for the management of liver fibrosis. Considering that the spontaneous activation of HSCs in culture is similar to that in vivo when fibrosis occurs [30,31], freshly isolated and culture-activated human primary HSCs were compared in our study.

## 2. Materials and Methods

### 2.1. Isolation, Culture, and Validation of Human Primary HSCs

**Figure 1:**
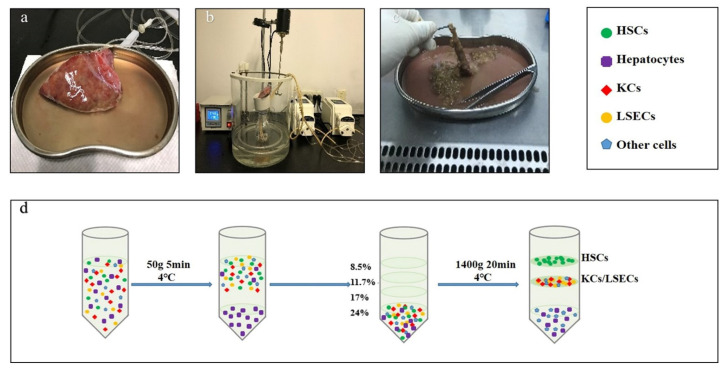
Isolation procedure of human primary HSCs from liver tissue specimens. (**a**) Perfusion catheters were inserted into the larger blood vessels, and the section of liver tissue was closed. (**b**) Liver tissue was digested by recirculating perfusion for 15–25 min using collagenase type IV. (**c**) The digested liver tissue was shredded with tweezers to prepare the cell suspension. (**d**) Hepatocytes were removed by centrifugation (50× *g*, 5 min, 4 °C, 3 times), and HSCs were isolated by density gradient centrifugation (1400× *g*, 20 min, 4 °C). Hepatic stellate cells, HSCs; Kupffer cells, KCs; liver sinusoidal endothelial cells, LSECs.

In our study, the digestion method using ethylene glycol tetraacetic acid (0.5 mM, Sigma, St. Louis, MO, USA)/collagenase type IV (0.8 mg/mL, Sigma) and density gradient centrifugation using Gey’s balanced salt solution (Sigma) and OptiprepTM (Axis-Shield, Oslo, Norway) was performed to isolate primary HSCs from human liver tissues, as described previously [32] (Figure 1). Samples were obtained from the abandoned tissue blocks of patients who had undergone liver transplantation or partial hepatectomy at the First Hospital of Jilin University (Changchun, China). Isolated human primary HSCs were cultured in Dulbecco’s modified Eagle’s medium (Gibco, Grand Island, NY, USA), supplemented with 10% fetal calf serum (Biological Industries, Kibbutz Beit-Haemek, Israel), at 37 °C in a 5% CO_2_ atmosphere. Culture-activated HSCs were harvested after 14 d of cultivation.

This study was approved by the ethics committee of the First Hospital of Jilin University (approval number 2018-408).

Autofluorescence of lipid droplets (LDs) (excitation: 328 nm) was performed to verify freshly isolated HSCs. The anti-α-smooth muscle actin (α-SMA) primary monoclonal antibody (1:500 dilution, Abcam, Cambridge, UK) and secondary polyclonal goat anti-rabbit IgG (Alexa Fluor^®^ 488) (1:1000 dilution; Abcam) were used for immunofluorescence staining of cultured HSCs, whereas 4’,6-diamidino-2-phenylindole was used to stain the nuclei. The HSC line LX-2 was used as a positive control.

### 2.2. RNA Isolation, Library Construction, and Sequencing

The total RNAs containing miRNAs were purified using TRIzol reagent (Invitrogen, Carlsbad, CA, USA). Agarose gel electrophoresis was performed to preliminarily monitor RNA degradation and contamination. RNA purity and integrity were measured with a NanoPhotometer^®^ spectrophotometer (IMPLEN, USA) and an Agilent Bioanalyzer 2100 system (Agilent Technologies, Palo Alto, CA, USA). Qualified RNA (~3 µg/sample) was used to construct a cDNA library, which was generated using the NEBNext^®^ Multiplex Small RNA Library Prep Set of Illumina^®^ (NEB, USA), per the manufacturer’s recommendations. Following amplification, targeted DNA fragments were recovered, and quality was assessed by the Agilent Bioanalyzer 2100 system using DNA high-sensitivity chips. Index codes were added in order to identify each sample. Clustering of index-coded samples was performed on a cBot Cluster Generation System using the TruSeq SR Cluster Kit v3-cBot-HS (Illumina, San Diego, IL, USA). Finally, the samples prepared by the library were sequenced using an Illumina Hiseq 2500 platform (Illumina), and 50 bp single-end reads were generated after cluster generation.

### 2.3. MiRNA-Seq Analysis

Quality control and read mapping were performed using the Bowtie software [33] to generate small RNA alignments. Subsequently, reads originating from protein-coding genes, repeat sequences, ribosomal RNAs, or other small RNAs were removed using RepeatMasker, the Rfam database, or species-specific data. Using the miRBase database as a reference, known and novel miRNAs were distinguished using miREvo [34], mirdeep2 [35], and sRNA-tools-cli. MiRNA expression was estimated by transcripts per million (TPM) using the normalization formula (normalized expression = mapped read count/total reads × 1,000,000). Pearson’s correlation and principal component analysis (PCA) were used to test the correlation of the expression profile and the clustering nature among the samples. Differential expression analysis between freshly isolated and culture-activated HSCs was performed using the DESeq2 method [36], based on the negative binomial distribution model. After screening for differentially expressed (DE) miRNAs, hierarchical clustering analysis was performed to determine the expression patterns of DEmiRNAs in different samples. In addition, our data were compared with open access miRNA-seq data derived from cirrhotic (*n* = 14) and healthy (*n* = 9) liver tissues [37].

### 2.4. Quantitative Real-Time PCR Analysis

Relative quantitative real-time PCR (qRT-PCR) was performed to confirm the expression of miRNAs associated with hepatic fibrosis in human primary HSCs. TransScript^®^ miRNA First-Strand cDNA Synthesis SuperMix (TransGen Biotech, Beijing, China) and PerfectStartTM Green qPCR SuperMix (TransGen Biotech) were used for the tailing reaction method. The PrimeScriptTMRT reagent Kit with gDNA Eraser (Takara, Tokyo, Japan) and TB Green^®^ Premix Ex TaqTM II (Takara) were used for the stem-loop method when reverse transcription and PCR were performed according to the manufacturer’s instructions. After 40–45 cycles on the Mx3005P system (Agilent Technologies), the relative expression of the selected miRNAs was calculated using the comparative cycle threshold method (2−ΔΔCt), with U6 as the endogenous control, to normalize the data. The primer sequences for the miRNAs used are listed in Supplementary Table S1.

### 2.5. Prediction of MiRNA-mRNA Interactions

The results from miRanda [38], PITA [39], and RNAhybrid [40] were intersected to predict the target genes of the miRNAs, in combination with the mRNA-seq data obtained by our team (Transcript Profiling: PRJNA762498). Pearson’s correlation was used to evaluate the tightness of miRNAs and their targeted mRNAs, including correlation coefficients and corresponding *p*-values. Subsequently, co-expression networks were constructed using Cytoscape [41]. Gene Ontology (GO) functional analysis and Kyoto Encyclopedia of Genes and Genomes (KEGG) pathway enrichment analysis were used to predict the target genes of DEmiRNAs using GOseq [42] and KOBAS [43].

### 2.6. Statistical Analyses

In miRNA-seq, demographic and clinical characteristics, as well as laboratory indices of patients, were expressed as the median (25th and 75th percentiles) or constituent ratios. *p*-values obtained by DESeq2 analysis were adjusted using the Benjamini and Hochberg method [44] to control the false discovery rate (FDR). A corrected *p*-value (defined as *q*) of 0.05 was set as the threshold for significant differential expression between the two groups. When the absolute value of Pearson’s correlation coefficient was ≥0.7 with *p* < 0.05, miRNAs and targeted mRNAs were considered to have potential functional interactions. In GO or KEGG enrichment analyses, *q* < 0.05 was selected as the differentially detected signal. A paired *t*-test was conducted to analyze the differences in miRNA expression from the qRT-PCR results, and * *p* < 0.05, ** *p* < 0.01, and **** *p* < 0.0001, from GraphPad Prism 8.0 (GraphPad Software, San Diego, CA, USA), were defined as statistically significant.

## 3. Results

### 3.1. Basic Characteristics of the Sequencing Subjects

MiRNA-seq analyses were conducted on human primary HSCs before and after in vitro culture (denoted by Group_Fre and Group_Act). HSCs were isolated from four patients (three men and one woman; the median age was 62 years) who underwent surgical procedures; detailed patient information is listed in Supplementary Table S2. The liver function of these patients was acceptable—aspartate aminotransferase and alanine aminotransferase levels were within the normal range, which minimized interference from intrahepatic inflammation in subsequent miRNA-seq results.

### 3.2. Acquisition of Human Primary HSCs

**Figure 2:**
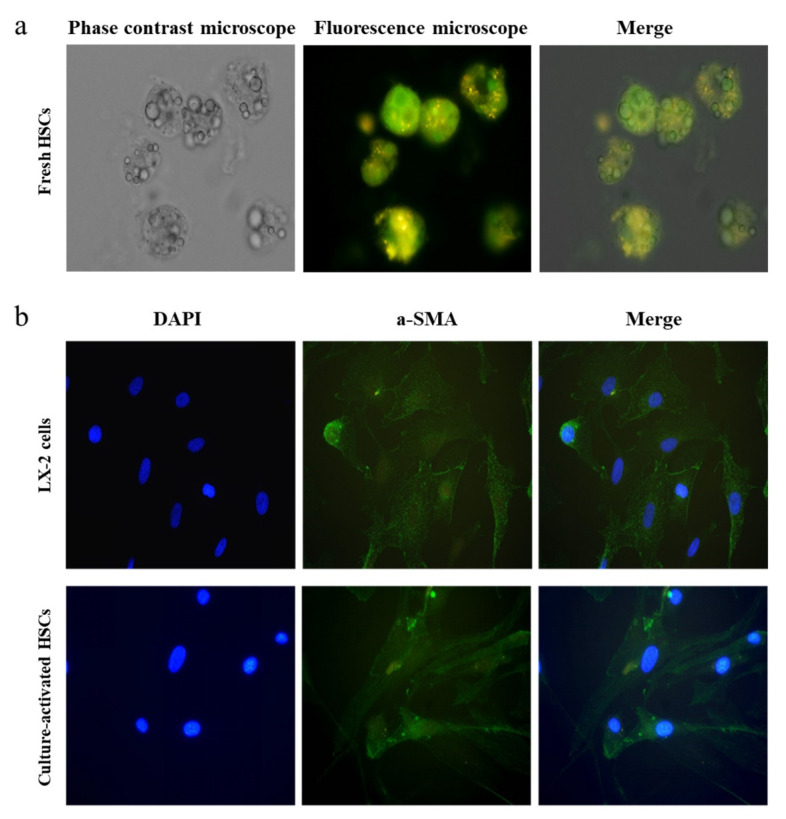
Identification of HSCs isolated from human liver tissues. (**a**) Autofluorescence of cytoplasmic vitamin A in freshly isolated HSCs. (**b**) Immunofluorescence staining of culture-activated HSCs and LX-2 cells for α-SMA (magnification: 400×; in (**a**,**b**)). α-smooth muscle actin, α-SMA; hepatic stellate cells, HSCs.

After repeated attempts, we successfully harvested 10^5^–10^6^ HSCs per gram of liver tissue; cell validation is shown in Figure 2. Freshly separated cells were round, small in volume, and rich in LDs (Figure 2a), with strong refraction. With extended incubation, HSCs became larger in volume and irregular in shape, with protrusions on the surface, loss of LDs, and deterioration of their refractive properties (Supplementary Figure S1a). Significant differences among individuals were observed when humans were the research object, and the activation process of HSCs was different. Generally, proliferation occurred after 7–10 days of culture, and HSCs were fully activated after 14 days of culture, along with a significant upregulation of α-SMA (Figure 2b), a specific biomarker of activated HSCs [3]. Therefore, 14 days was selected as the cutoff point for subsequent studies.

### 3.3. MiRNA Profiling

**Figure 3:**
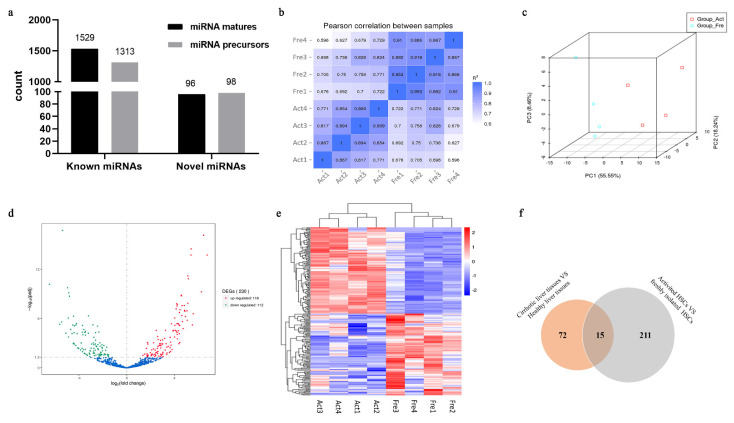
Evaluation of sequencing data and identification of candidate DEmiRNAs. (**a**) The number of miRNA matures and precursors from sequencing. (**b**) Pearson correlation heat-map of miRNA expression in different samples. R^2^—The square of Pearson’s correlation coefficients. (**c**) PCA plot between groups. Each point represents one sample. The red square represents a sample from Group_Act and the blue circle represents a sample from Group_Fre. (**d**) Volcano plot of miRNAs differentially expressed between Group_Fre and Group_Act. Each point represents one detectable miRNA. (**e**) Cluster of 230 DEmiRNAs between groups. Log10 (TPM + 1) values were used for clustering. (**f**) A Venn diagram of miRNA-seq data for HSCs and liver tissues. Four novel miRNAs in our data were not considered. Differentially expressed microRNAs, DEmiRNAs; principal component analysis, PCA; hepatic stellate cells, HSCs; microRNA sequencing, miRNA-seq; versus, VS.

An assessment of miRNA integrity and purity was performed before sequencing, and the results met the requirements for database construction (Supplementary Table S3). The data quality produced by small RNA sequencing demonstrated that the overall error rate for each sample ranged from 0.00% to 0.01%, and Q30 values were over 98% (Supplementary Table S4), a prerequisite for effective downstream analyses. An average of 93.63% of small RNAs were mapped to the human genome. From the mapped miRNA sequences, 1529 known matures and 1313 known precursors were obtained, and 96 novel matures and 98 novel precursors were predicted after excluding coding sequences and their degradation products, repetitive sequences, ribosomal RNAs, and other small RNAs ( ). TPM values were used to estimate the normalized transcription levels of miRNAs. The correlation matrix and PCA plot indicated high consistency and clustering within each group, as well as significant differences before and after HSC culture, suggesting that miRNAs were significantly altered by HSC activation (Figure 3b,c). Expression levels of *COL1A1*, *COL1A2*, and *ACTA2* in the four pairs of samples used for miRNA-seq are shown in Supplementary Figure S1b, in order to demonstrate the activation status of HSCs.

### 3.4. Identification of DEmiRNAs

We identified 230 DEmiRNAs, including 226 known and 4 novel miRNAs, per the absolute value of log_2_ fold change (FC) set at greater than or equal to 1.0 (|log2 FC| ≥ 1.0), and that of *q* set at <0.05 (Supplementary Table S5). During HSC activation in vitro, the expression of 118 and 112 miRNAs was significantly upregulated and downregulated, respectively (Figure 3d). Hierarchical clustering of DEmiRNAs revealed that the expression patterns of DEmiRNAs among the samples in the same group were similar, but changed after HSC activation (Figure 3e).

In order to search for key candidate miRNAs, we focused on miRNAs with the most significant expression differences (|log_2_ FC| > 5.0 and *q* < 0.05; Table 1); most of them (70.59%) were significantly upregulated. Through a search of the literature, *miR-758-3p*, *miR-493-5p*, *miR-409-3p*, *miR-31-5p*, *miR-1268a*, *miR-381-3p*, *miR-127-5p*, *miR-375-3p*, and *miR-548ah-5p* were identified as closely related to liver diseases [45,46,47,48,49,50,51,52,53,54,55,56,57], and they mainly play a role in HCC. Except for *miR-375-3p* [56], the relationship between these miRNAs and hepatic fibrosis has not been elucidated. Therefore, our study was carried out with special attention to these miRNAs.

We compared our data with open access miRNA-seq data from 2018, from 14 cirrhotic and 9 healthy liver tissue samples [37]. Comparison with 87 DEmiRNAs in the published results identified 15 overlapping DEmiRNAs (17.24%), including *let-7g-5p*, *miR-101-3p*, *miR-107*, *miR-122-5p*, *miR-127-3p*, *miR-139-5p*, *miR-148a-3p*, *miR-192-5p*, *miR-194-5p*, *miR-215-5p*, *miR-24-3p*, *miR-26a-5p*, *miR-340-5p*, *miR-451a*, and *miR-99a-5p*, suggesting that they are related to hepatic fibrosis in both studies (Figure 3f). To be consistent with the published data, log_2_FC values of DEmiRNAs in our study were converted to FC. A total of 12 miRNAs (80%) had a concordant expression pattern in the two comparison schemes; the same miRNA was upregulated or downregulated in liver cirrhosis and activation of HSCs (Table 2). However, the expression of *miR-101-3p*, *miR-192-5p*, and *miR-24-3p* was contradictory between the two datasets.

**Figure 4:**
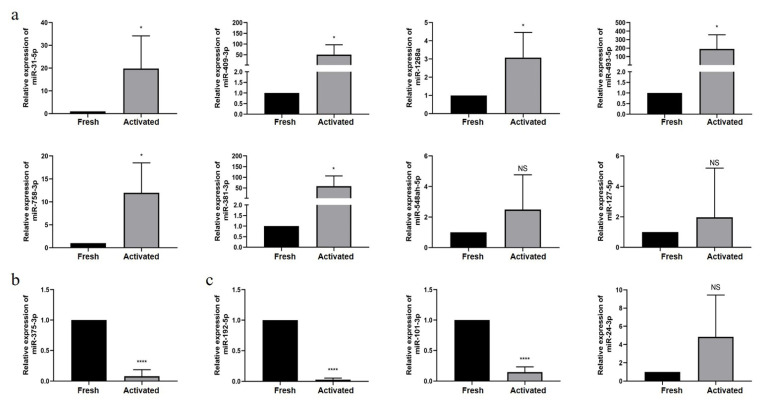
QRT-PCR validation of the expression of miRNAs. QRT-PCR validation of the expression of (**a**) miRNAs associated with liver diseases, (**b**) *miR-375-3p*, the miRNA associated with cirrhosis, and (**c**) miRNAs with contradictory expression. * *p* < 0.05, **** *p* < 0.0001, and NS, *p* ≥ 0.05 versus freshly isolated HSCs.

In order to further validate the selected DEmiRNAs, qRT-PCR was performed. When HSCs were activated in vitro, *miR-375-3p*, a hepatic fibrosis-related miRNA, was significantly downregulated (*p* < 0.0001) (Figure 4b), in accordance with a previous study [56]. As expected from miRNA-seq, the expression of miRNAs associated with liver diseases, such as *miR-409-3p*, *miR-31-5p*, *miR-1268a*, *miR-758-3p*, *miR-493-5p*, and *miR-381-3p*, was confirmed to be significantly increased compared to that in the control group (*p* < 0.05). However, no significant difference in the expression of *miR-127-5p* or *miR-548ah-5p* was observed (Figure 4a). Regarding the common miRNAs with contradictory expression results between the two datasets, *miR-101-3p* and *miR-192-5p* were significantly downregulated in activated HSCs (*p* < 0.0001), supporting our miRNA-seq data. Although *miR-24-3p* was upregulated, this was not statistically significant (Figure 4c).

### 3.5. Target Prediction and Co-Expression Analysis

**Figure 5:**
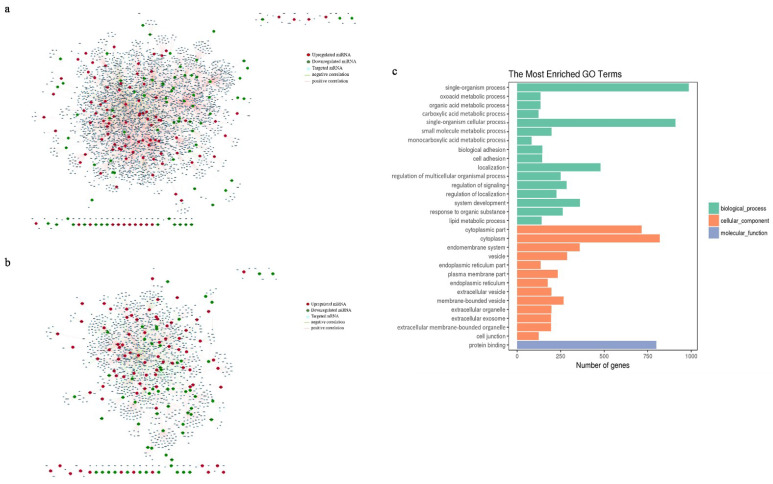
Construction of the co-expression networks for miRNAs–mRNAs and functional analysis of targeted genes. Construction of co-expression networks for (**a**) DEmiRNAs–mRNAs and (**b**) DEmiRNAs–DEmRNAs. Data with |Pearson’s correlation coefficient| ≥ 0.7 and *p* < 0.05 are shown in the networks. Red, green, and blue dots represent significantly upregulated miRNAs, significantly downregulated miRNAs, and targeted mRNAs, respectively. The pink and green lines represent positive and negative correlations, respectively. (**c**) Top 30 GO terms in the GO enrichment analysis of targeted DEmRNAs. Green, orange, and purple represent BP, CC, and MF, respectively. MicroRNA, miRNA; differentially expressed, DE; biological process, BP; cellular component, CC; molecular function, MF.

Since miRNAs play a biological role by targeting mRNAs, we combined the data from this study with previous mRNA data from our team (Transcript Profiling: PRJNA762498) and predicted and evaluated target mRNAs using several software packages and screening conditions (Supplementary Table S6). We constructed miRNA–mRNA co-expression networks using Cytoscape. Figure 5a illustrates the relationship between all DEmiRNAs and their target genes. Of the 230 DEmiRNAs, 175 (76.1%) were strongly correlated with their corresponding target mRNAs. The network map between DEmiRNAs and target DEmRNAs when the differential expression information of mRNAs (*q* < 0.05) is also taken into account is displayed in Figure 5b. Ultimately, the network consisted of 1891 miRNA–mRNA pairs, containing 138 DEmiRNAs and corresponding to 1414 DEmRNAs (1193 genes). *MiR-665* exhibited the most correlations (208 targets).

Target genes of liver disease-related miRNAs that showed significant differences were confirmed by qRT-PCR analysis and are summarized in Table 3. *MiR-1268a* had the most targets, indicating a potentially important role in the regulation of mRNA expression, which might affect the development of fibrosis. Thereafter, we attempted to transfect the human HSC line (LX-2) with miRNA mimics or inhibitors in order to observe their effect on cells. Prior results indicated that *COL1A1* expression was significantly upregulated after transfection with the *miR-1268a* inhibitor or *miR-665* mimic (Supplementary Table S7 and Supplementary Figure S2).

In order to explore the function of miRNAs, we performed GO functional and KEGG pathway enrichment analyses. The cellular component (CC), molecular function (MF), and biological process (BP) of the targeted genes (only DEmRNAs were included) were described in the GO analysis. We found that the DEmRNAs were significantly enriched in 417 terms (BP: 308 terms; CC: 74 terms; MF: 35 terms; *q* < 0.05; Figure 5c). In the CC category, the most enriched GO terms were involved in the cytoplasm, plasma membrane, extracellular region, and other components; in the MF category, DEmRNAs were significantly enriched in “cytoskeletal protein binding”, “cell adhesion molecule binding”, “ECM structural constituent”, “ion binding”, “catalytic activity”, and “molecular function regulator”; and in the BP category, “cell adhesion”, “organic acid metabolism”, “lipid metabolism”, and “collagen metabolism” were the most significant GO terms. KEGG analysis revealed that DEmRNAs were engaged in 262 pathways, such as tyrosine metabolism and complement and coagulation cascades. However, no significantly enriched pathways were found, since dispersed pathways were involved.

## 4. Discussion

In end-stage conditions, decompensated hepatocirrhosis and HCC are the main causes of death in patients with hepatic fibrosis [58,59]. However, the relevant mechanisms remain unelucidated. The activation and proliferation of HSCs is a dominant event in hepatic fibrosis [2,60]; therefore, studies specific to HSCs are needed to elucidate the underlying critical events in the progression of this disease. Endogenous miRNAs, originally considered “junk” substances, have fundamental effects on the regulation of physiological and pathological processes, and have a strong predictive value for the assessment and diagnosis of numerous diseases [61,62,63]. Unlike animal models of cirrhosis, our study used clinical samples to determine the miRNA expression profile in human primary HSCs using NGS technology. High-throughput sequencing of miRNAs enabled us to identify previously overlooked miRNAs involved in hepatic fibrosis.

To a certain extent, HSCs appear to be “activated” spontaneously after in vitro culture and develop a fibroblastic morphology, which has much in common with the response of HSCs in the occurrence of hepatic fibrosis caused by chronic liver damage [30,31,64]. Moreover, the gene expression pattern of HSCs could be altered after a short period of culture. Comparative analysis of human primary HSCs before and after activation was an attempt to simulate the disease process, thereby minimizing the variability of experimental results due to differences in the etiology, disease stage, or collection procedure of samples. Meanwhile, a matched-pair analysis avoided the impacts of individual differences and provided reliable data for the purpose of accurately identifying changes in diseases.

In this study, the 230 selected DEmiRNAs included four novel miRNAs; however, their properties are unclear. The purpose of this study was to identify the unknown characteristics and functions of known miRNAs. In addition to KEGG analysis and the protein–protein interaction network, comparison of FC values used to measure gene expression was a convenient method to explore candidate biomarkers. After in vitro activation of HSCs, several miRNAs were significantly upregulated or downregulated, suggesting that they may promote or interfere with HSC activation, thus influencing fibrogenesis. Thereafter, we highlighted a subset of these miRNAs.

Related studies have implicated *miR-758-3p*, *miR-381-3p*, *miR-31-5p*, *miR-493-5p*, *miR-1268a*, and *miR-127-5p* in HCC. Overexpression of these miRNAs suppressed the proliferation and invasion of HCC cells by targeting the corresponding genes [45,46,47,48,49,50,55]. Using network analysis, Tang et al. [52] found that *miR-381-3p* may be a core regulatory molecule in HCC, and Lu et al. [54] reported that a low expression of *miR-1268a* is an independent risk factor for HCC prognosis. *MiR-548* family members are associated with impaired interferon signaling in chronic HBV infection [57]. *MiR-409-3p* was upregulated in mice with non-alcoholic fatty liver disease (NAFLD), and might be a key regulator of NAFLD progression as well as a non-invasive diagnostic indicator of NAFLD severity and progression [51].

Although these eight miRNAs have been previously described in the study of various liver diseases, they are not known to be involved in hepatic fibrosis; therefore, we selected them for subsequent qRT-PCR verification. PCR results suggested that following HSC activation, the differential expression of six liver disease-related miRNAs, including *miR-409-3p*, *miR-31-5p*, *miR-1268a*, *miR-758-3p*, *miR-493-5p,* and *miR-381-3p*, was consistent with the transcriptome analysis. Therefore, these miRNAs might also have certain effects on hepatic fibrosis. Yang et al. [56] found that a high fructose intake reduced the expression of *miR-375-3p*, activated the TGF-β/Smad signaling pathway, and induced epithelial-mesenchymal transition and liver fibrosis. Similarly, our findings showed that *miR-375-3p* was downregulated when HSCs were activated.

Our study focused on HSCs cultured in vitro, and lacked comparison between normal and cirrhotic livers. We considered published data in order to fill this gap. Koduru et al. [37] reported significant differences in the expression of 87 miRNAs in cirrhotic liver tissues compared with normal tissues, of which 15 miRNAs also appeared in our list of DEmiRNAs. After integrating the two gene expression profile datasets, we found that it was more reliable to use commonly altered miRNAs as candidate biomarkers for the diagnosis of hepatic fibrosis. The expression patterns of *miR-101-3p*, *miR-192-5p*, and *miR-24-3p* were contradictory in the two datasets, which could be attributed to inaccurate sequencing or differences between HSC activation in cirrhotic liver tissues, both in vivo and in vitro. Furthermore, *miR-101-3p* was downregulated in the human HSC line LX-2, and its overexpression could inhibit the proliferation and migration of LX-2 cells [65], supporting the findings of our study.

In contrast to siRNAs, miRNAs, as endogenous transcriptional fragments, target mRNAs and influence the expression of coding genes [66]. The prediction and enrichment analyses of target genes could be of considerable significance for studying the function of miRNAs that have not yet been implicated in fibrosis, and the description of the co-expression regulatory network could also provide additional information. In general, “|Pearson’s correlation coefficient| > 0.7” is considered a strong correlation between variables, and the reliability of target genes is high under such conditions. Collectively, 138 DEmiRNAs were potentially bound to 1193 DE genes, which were involved in different types of collagen (*COL4A1*, *COL4A2*, *COL13A1*, *COL14A1*, *COL16A1*, and *COL26A1*), *lysyl oxidase-like 2* (*LOXL2*) [67], *platelet-derived growth factor receptor β* (*PDGFRβ*) [68], and other fibrosis-related genes. They participated in biological processes, such as collagen metabolism, ECM structural composition, cytoskeleton protein binding, and cell adhesion, suggesting that these DEmiRNAs are associated with critical paths of fibrosis. In addition, retinoid X receptor α (RXRα) is an important nuclear receptor that plays a key regulatory role in liver fibrosis [69,70]. From the miRNA–mRNA interaction network, *miR-654-5p* was predicted to target *RXRα*, which has not been reported before. We also found that *miR-654-5p* was significantly upregulated, while *RXRα* expression was downregulated in activated HSCs (from mRNA-seq data). On the basis of the above results, our team elucidated that *miR-654-5p* aggravated liver fibrosis by blocking RXRα [71]. Among the numerous miRNAs, *miR-665* and *miR-1268a* had the highest regulatory associations, and might be promising markers for hepatic fibrosis. Coincidentally, a recent study showed that the high expression of *miR-665* contributed to larger tumor volume and more severe vascular invasion and pathological grade in patients with HCC [72].

During fibrosis, the expression of miRNAs can be upregulated or downregulated. Upregulated miRNAs could be reversed by miRNA-masking antisense oligonucleotide technology, whereas downregulated expression could be restored by transfection of miRNA mimics, plasmids, or viral vectors [73,74]. In our study, transfection with miRNA mimics or inhibitors was used to artificially interfere with the role of miRNAs, and the effects of *miR-1268a* and *miR-665* on hepatic fibrosis were preliminarily analyzed. In LX-2 cells, *miR-1268a* silencing or *miR-665* overexpression elevated *COL1A1* mRNA levels.

Overall, we gained a deeper insight into miRNA regulation in hepatic fibrosis, but some limitations exist in this study. First, previous assumptions were based on animal experiments or cell lines, with no or limited evidence from human studies. Because human tissues are difficult to obtain, the number of samples included was small, and the comparison between normal and cirrhotic livers was lacking. Second, using transcriptome analysis, we preliminarily identified some miRNAs that might have important effects on HSC activation; however, their specific functions and pathways need to be explored.

## 5. Conclusions

This study presents the expression information of miRNAs during the activation of human HSCs and potential miRNA–mRNA co-expression networks using NGS. Our findings provide a clear direction for the study of the specific mechanism of miRNAs in liver fibrosis, which could yield novel potential diagnostic biomarkers or therapies for patients with liver fibrosis or cirrhosis.

## Figures and Tables

**Table 1:** DEmiRNAs in culture-activated HSCs versus freshly isolated HSCs (|log2fold change| > 5 and *q* < 0.05).

MiRNAs	Log_2_fold Change	*p*	*q*	Associated with Liver Disease or not (from Pubmed)
*miR-758-3p*	6.74	1.22 × 10^16^	1.89 × 10^14^	HCC
*miR-493-5p*	6.43	1.88 × 10^19^	7.28 × 10^17^	HCC
*miR-31-3p*	6.25	7.30 × 10^12^	5.66 × 10^10^	-
*miR-376a-3p*	5.58	9.45 × 10^9^	2.71 × 10^7^	-
*miR-1293*	5.50	3.65 × 10^7^	6.02 × 10^6^	-
*miR-654-5p*	5.40	1.29 × 10^17^	3.34 × 10^15^	-
*miR-31-5p*	5.38	1.19 × 10^16^	1.89 × 10^14^	HCC
*miR-495-3p*	5.17	1.31 × 10^13^	1.46 × 10^11^	-
*miR-409-3p*	5.12	5.13 × 10^14^	6.63 × 10^12^	NAFLD
*miR-381-3p*	5.12	2.72 × 10^10^	1.40 × 10^8^	HCC
*miR-1268a*	5.07	7.03 × 10^8^	1.60 × 10^6^	HCC
*miR-127-5p*	5.04	3.70 × 10^9^	1.19 × 10^7^	HCC
*miR-1295a*	−5.09	5.65 × 10^6^	6.02 × 10^5^	-
*miR-375-3p*	−5.38	2.39 × 10^20^	1.85 × 10^17^	Hepatic fibrosis and HCC
*miR-1295b-3p*	−5.46	1.21 × 10^7^	2.60 × 10^6^	-
*miR-548ah-5p*	−5.61	2.72 × 10^7^	4.91 × 10^6^	CHB
*novel_365*	−6.47	7.35 × 10^13^	7.12 × 10^11^	-

The literature retrieval included results published until the middle of 2020. MicroRNAs, miRNAs; non-alcoholic fatty liver disease, NAFLD; hepatocellular carcinoma, HCC; chronic hepatitis B, CHB.

**Table 2:** Overlapping DEmiRNAs with concordant expression between HSCs’ miRNA-seq data and liver tissues’ miRNA-seq data.

MiRNAs	HSCs’ miRNA-Seq Data		Liver Tissues’ MiRNA-seq Data *	
	Fold Change	*q* Value	Fold Change	*q* Value
*let-7g-5p*	−3.76	1.90 × 10^3^	−204.40	2.12 × 10^4^
*miR-107*	−5.94	2.20 × 10^3^	−2.62	1.55 × 10^2^
*miR-122-5p*	−18.51	4.12 × 10^6^	−312.73	1.58 × 10^8^
*miR-127-3p*	18.64	3.01 × 10^8^	17.15	3.85 × 10^4^
*miR-139-5p*	−9.32	6.02 × 10^5^	−14.07	4.82 × 10^3^
*miR-148a-3p*	−10.93	3.65 × 10^5^	−10.94	2.52 × 10^4^
*miR-194-5p*	−21.86	5.35 × 10^7^	−1.86	2.04 × 10^2^
*miR-215-5p*	−7.16	5.79 × 10^6^	−3.07	4.17 × 10^2^
*miR-26a-5p*	−4.20	1.60 × 10^3^	−3.79	5.34 × 10^3^
*miR-340-5p*	−6.11	5.10 × 10^5^	−4.04	9.41 × 10^3^
*miR-451a*	−4.86	2.22 × 10^2^	−62.96	3.56 × 10^4^
*miR-99a-5p*	−6.96	9.00 × 10^4^	−12.34	1.77 × 10^4^

* Data were cited from a published study, DOI:10.1038/s41598-018-26360-1. MicroRNAs, miRNAs; hepatic stellate cells, HSCs.

**Table 3:** The number of mRNAs targeted by miRNAs.

MiRNAs	Number of Target mRNAs	Number of Target DEmRNAs
*miR-758-3p*	2	1
*miR-493-5p*	3	1
*miR-31-5p*	23	13
*miR-409-3p*	1	1
*miR-381-3p*	3	2
*miR-1268a*	125	47
*miR-375-3p*	0	0

Target mRNAs with |Pearson’s correlation coefficient| ≥ 0.7 and *p* < 0.05 were counted in the table. MicroRNAs, miRNAs; differentially expressed mRNAs, DEmRNAs.

## Data Availability

The datasets generated and/or analyzed during the current study will not be publicly available for four years (SRA number: PRJNA753987) due to our intention to conduct further research, but are available from the corresponding author upon reasonable request.

## Supplementary Materials

The following supporting information can be downloaded at: https://www.mdpi.com/article/10.3390/genes13122201/s1, Figure S1: The characteristics of activated HSCs. (a) Cell morphology of HSCs cultured in vitro for 14 days as shown in a light microscopic field (original magnification: 100×). (b) Expression levels of liver fibrosis-related genes in four pairs of HSCs used for miRNA-seq. The results are derived from mRNA-seq data obtained by our team (Transcript Profiling: PRJNA762498); Figure S2: Effects of miR-1268a and miR-665 on the expression of COL1A1. Expression of COL1A1 mRNA after transfection of (a) miR-1268a inhibitor and (b) miR-665 mimic. negative control, NC.; Table S1: Primer sequences of miRNAs for qRT-PCR; Table S2: Basic characteristics of patients in miRNA-seq; Table S3: Purity and integrity of RNAs for miRNA-seq; Table S4: Quality assessment of small RNA sequencing data; Table S5: The list of 230 DEmiRNAs; Table S6: The list of target DEmRNAs; Table S7: Sequences of RNAs for transfection and primer sequences of miRNAs for qRT-PCR.
