# Supplementary material for: Identification of Liver Fibrosis-Related MicroRNAs in Human Primary Hepatic Stellate Cells Using High-Throughput Sequencing
Source: Genes (Basel). 2022 Nov 24;13(12):2201. doi: 10.3390/genes13122201 (PMC9778123; doi:10.3390/genes13122201)
Supplement: Supplementary file 1 [file genes-13-02201-s001.zip › Supplementary Figure S1.pdf]

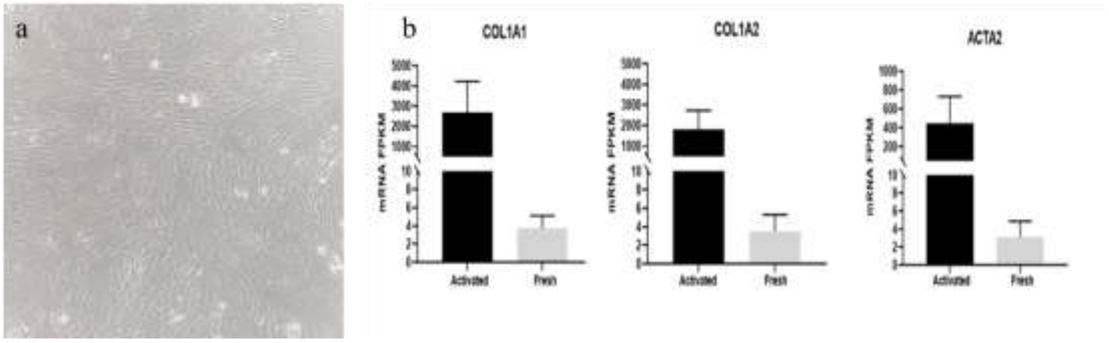

Supplementary Figure S1. The characteristics of activated HSCs. (a) Cell morphology of HSCs cultured in *vitro* for 14 days as shown in a light microscopic field (original magnification: 100×). (b) Expression levels of liver fibrosis-related genes in four pairs of HSCs used for miRNA-seq. The results are derived from mRNA-seq data obtained by our team (Transcript Profiling: PRJNA762498).
