# Supplementary material for: Identification of Liver Fibrosis-Related MicroRNAs in Human Primary Hepatic Stellate Cells Using High-Throughput Sequencing
Source: Genes (Basel). 2022 Nov 24;13(12):2201. doi: 10.3390/genes13122201 (PMC9778123; doi:10.3390/genes13122201)
Supplement: Supplementary file 1 [file genes-13-02201-s001.zip › Supplementary Figure S2.pdf]

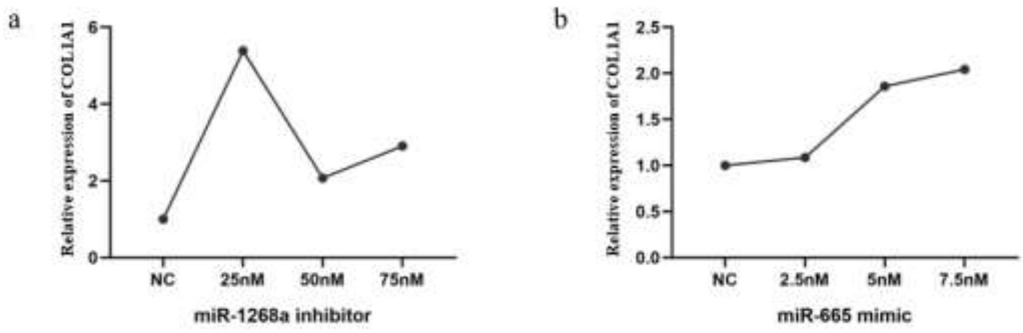

Supplementary Figure S2. Effects of miR-1268a and miR-665 on the expression of COL1A1. Expression of COL1A1 mRNA after transfection of (a) miR-1268a inhibitor and (b) miR-665 mimic. negative control, NC.
