# Supplementary material for: Identification of Liver Fibrosis-Related MicroRNAs in Human Primary Hepatic Stellate Cells Using High-Throughput Sequencing
Source: Genes (Basel). 2022 Nov 24;13(12):2201. doi: 10.3390/genes13122201 (PMC9778123; doi:10.3390/genes13122201)
Supplement: Supplementary file 1 [file genes-13-02201-s001.zip › Supplementary Table S1.pdf]

Supplementary Table S1. Primer sequences of miRNAs for qRT-PCR

MiRNAs	Primer sequences	
**MiRNAs for stem-loop method**		
U6	Forward	5′-AGAGAAGATTAGCATGGCCCCTG-3′
U6	RT Primer	5′-GTCGTATCCAGTGCAGGGTCCGAGGTAT TCGCACTGGATACGACAAAATA-3′
miR-548ah-5p	Forward	5′-CGCGCGAAAAGTGATTGCAG-3′
miR-548ah-5p	RT Primer	5′-GTCGTATCCAGTGCAGGGTCCGAGGTAT TCGCACTGGATACGACCAAACA-3′
miR-381-3p	Forward	5′-CGCGTATACAAGGGCAAGCT-3′
miR-381-3p	RT Primer	5′-GTCGTATCCAGTGCAGGGTCCGAGGTAT TCGCACTGGATACGACACAGAG-3′
miR-758-3p	Forward	5′-GCGTTTGTGACCTGGTCCA-3′
miR-758-3p	RT Primer	5′-GTCGTATCCAGTGCAGGGTCCGAGGTAT TCGCACTGGATACGACGGTTAG-3′
miR-24-3p	Forward	5′-GCGTGGCTCAGTTCAGCAG-3′
miR-24-3p	RT Primer	5′-GTCGTATCCAGTGCAGGGTCCGAGGTAT TCGCACTGGATACGACCTGTTC-3′
miR-192-5p	Forward	5′-GCGCGCTGACCTATGAATTG-3′
miR-192-5p	RT Primer	5′-GTCGTATCCAGTGCAGGGTCCGAGGTAT TCGCACTGGATACGACGGCTGT-3′

miR-101-3p	Forward	5′-GCGCGCGTACAGTACTGTGATA-3′
miR-101-3p	RT Primer	5′-GTCGTATCCAGTGCAGGGTCCGAGGTAT TCGCACTGGATACGACTTCAGT-3′
miR-409-3p	Forward	5′-CGGAATGTTGCTCGGTGA-3′
miR-409-3p	RT Primer	5′-GTCGTATCCAGTGCAGGGTCCGAGGTAT TCGCACTGGATACGACAGGGGT-3′
miR-493-5p	Forward	5′-CGCGTTGTACATGGTAGGCT-3′
miR-493-5p	RT Primer	5′-GTCGTATCCAGTGCAGGGTCCGAGGTAT TCGCACTGGATACGACAATGAA-3′
miR-375-3p	Forward	5′-GCGTTTGTTCGTTCGGCTC-3′
miR-375-3p	RT Primer	5′-GTCGTATCCAGTGCAGGGTCCGAGGTAT TCGCACTGGATACGACTCACGC-3′
**MiRNAs for tailing reaction method**		
U6	Forward	5′-CCTGGCACCCAGCACAAT-3′
miR-1268a	Forward	5′-GCGTGGTGGTGGGGGAA-3′
miR-665	Forward	5′-GGAGGCTGAGGCCCCTAAA-3′
miR-31-5p	Forward	5′-GCAAGATGCTGGCATAGCTAA-3′
miR-127-5p	Forward	5′-TGAAGCTCAGAGGGCTCTGATAA-3′

The reverse primer sequences of miRNAs are universal, which are kept secret by the company.

microRNAs, miRNAs; quantitative real-time PCR, qRT-PCR.
