# Supplementary material for: Identification of Liver Fibrosis-Related MicroRNAs in Human Primary Hepatic Stellate Cells Using High-Throughput Sequencing
Source: Genes (Basel). 2022 Nov 24;13(12):2201. doi: 10.3390/genes13122201 (PMC9778123; doi:10.3390/genes13122201)
Supplement: Supplementary file 1 [file genes-13-02201-s001.zip › Supplementary Table S2.pdf]

Supplementary Table S2. Basic characteristics of patients in miRNA-seq

Variables	Basic characteristics of patients (n=4)
**Demographic characteristics**	
Age (years)	62.00(56.00, 66.50)
Sex, male[n (%)]	3(75)
**Clinical characteristics**	
AST (IU/L)	26.85(19.85, 35.95)
ALT (IU/L)	23.40(20.63, 26.25)
TBIL (umol/L)	17.90(10.03, 21.88)
Albumin (g/L)	37.90(32.43, 43.15)
Cholinesterase (U/L)	3859.00(2988.75, 6744.50)
INR	1.15(0.96, 1.35)
**Laboratory indices**	
Warm ischemia time (min)	49.00(38.50, 78.25)
Tissue weight (g)	44.00(36.25, 86.25)
Yield of HSCs(/g)	$3.01*10^5(1.01*10^5, 7.23*10^6)$

Data were expressed as median (25th, 75th percentiles) or percentage.

hepatic stellate cells, HSCs; aspartate aminotransferase, AST; alanine aminotransferase, ALT; total bilirubin, TBIL; international normalized ratio, INR.
