# Supplementary material for: Identification of Liver Fibrosis-Related MicroRNAs in Human Primary Hepatic Stellate Cells Using High-Throughput Sequencing
Source: Genes (Basel). 2022 Nov 24;13(12):2201. doi: 10.3390/genes13122201 (PMC9778123; doi:10.3390/genes13122201)
Supplement: Supplementary file 1 [file genes-13-02201-s001.zip › Supplementary Table S3.pdf]

Supplementary Table S3. Purity and integrity of RNAs for miRNA-seq

Sample names*	OD260/280	OD260/230	28S/18S	RIN
Fre1	1.95	1.87	1.10	7.50
Fre2	1.89	1.02	1.50	8.50
Fre3	1.98	1.38	1.40	9.10
Fre4	1.99	1.92	1.20	9.20
Act1	2.23	0.78	2.60	8.30
Act2	2.50	0.52	2.10	7.70
Act3	2.00	1.96	2.60	10.00
Act4	2.10	1.57	3.20	10.00

*The samples named Fre1-Fre4 came from Group_Fre and the samples named Act1-Act4 came from Group_Act. The samples with the same serial number are the paired samples.
