# Supplementary material for: Identification of Liver Fibrosis-Related MicroRNAs in Human Primary Hepatic Stellate Cells Using High-Throughput Sequencing
Source: Genes (Basel). 2022 Nov 24;13(12):2201. doi: 10.3390/genes13122201 (PMC9778123; doi:10.3390/genes13122201)
Supplement: Supplementary file 1 [file genes-13-02201-s001.zip › Supplementary Table S4.pdf]

Supplementary Table S4. Quality assessment of small RNA sequencing data

Sample names*	Raw reads	Error rate(%)	Q20(%)	Q30(%)	GC content(%)
Fre1	$1.15*10^7$	0.01	99.78	99.36	49.84
Fre2	$1.04*10^7$	0.00	99.84	99.54	48.88
Fre3	$1.15*10^7$	0.01	99.73	99.22	51.21
Fre4	$1.50*10^7$	0.00	99.83	99.50	50.03
Act1	$1.54*10^7$	0.01	99.39	98.26	49.89
Act2	$1.44*10^7$	0.01	99.62	98.97	49.88
Act3	$1.25*10^7$	0.00	99.81	99.48	51.01
Act4	$1.41*10^7$	0.01	99.73	99.25	51.66

*The samples named Fre1-Fre4 came from Group_Fre and the samples named Act1-Act4 came from Group_Act. The samples with the same serial number are the paired samples.
