# Supplementary material for: Identification of Liver Fibrosis-Related MicroRNAs in Human Primary Hepatic Stellate Cells Using High-Throughput Sequencing
Source: Genes (Basel). 2022 Nov 24;13(12):2201. doi: 10.3390/genes13122201 (PMC9778123; doi:10.3390/genes13122201)
Supplement: Supplementary file 1 [file genes-13-02201-s001.zip › Supplementary Table S7.pdf]

Supplementary Table S7. Sequences of RNAs for transfection and primer sequences of miRNAs for qRT-PCR.

RNAs	Sequences*	
**For transfection**		
miR-1268a inhibitor	5′-CCCCCACCACCACGCCCG-3′	
inhibitor NC	5′-CAG UAC UUU UGU GUA GUA CAA-3′	
miR-665 mimic	Sense	5′-ACCAGGAGGCUGAGGCCCCU-3′
miR-665 mimic	Antisense	5′-AGGGGCCUCAGCCUCCUGGU-3′
mimic NC	Sense	5′-UUC UCC GAA CGU GUC ACG UTT-3′
mimic NC	Antisense	5′-ACG UGA CAC GUU CGG AGA ATT-3′
**For qRT-PCR**		
β-actin	Forward	5′-CCTGGCACCCAGCACAAT-3′
β-actin	Reverse	5′-GGGCCGGACTCGTCATAC-3′
COL1A1	Forward	5′-GAGGGCCAAGACGAAGACATC-3′
COL1A1	Reverse	5′-CAGATCACGTCATCGCACAAC-3′

*The miRNA inhibitor is single stranded and the miRNA mimic is made up of double strands.

negative control, NC; quantitative real time PCR, qRT-PCR.
